# Measure quantity of mitochondrial DNA in aged bones or calculate it from nuclear DNA quantitative PCR results?

**DOI:** 10.1007/s00414-023-03074-2

**Published:** 2023-08-10

**Authors:** Marcel Obal, Tomaž Zupanc, Irena Zupanič Pajnič

**Affiliations:** Institute of Forensic Medicine, Faculty of Medicine, University of Ljubljana, Korytkova 2, 1000 Ljubljana, Slovenia

**Keywords:** qPCR, mtDNA, nDNA, Skeletal remains, Second World War

## Abstract

Mitochondrial DNA (mtDNA) is of great value in forensics to procure information about a person when a next of kin, personal belongings, or other sources of nuclear DNA (nDNA) are unavailable, or nDNA is lacking in quality and quantity. The quality and reliability of the results depend greatly on ensuring optimal conditions for the given method, for instance, the optimal input of the copy number (CN) in next-generation sequencing (NGS) methods. The unavailability of commercial quantitative PCR (qPCR) methods to determine mtDNA CN creates the necessity to rely on recommendations to infer mtDNA CN from nDNA yield. Because nDNA yield varies between individuals, tissues, parts of the same tissue, and because mtDNA CN varies between tissues, such assumptions must be examined for a specific context, rather than be generalized. This study compares mtDNA CN calculated from nDNA yield and qPCR measured mtDNA CN. Seventy-five femurs from the Second World War victims were used as samples; they were cut below the greater trochanter, surface contaminants were removed by mechanical and chemical cleaning, samples were fully demineralized, and DNA was isolated. PowerQuant® Kit (Promega) was used to analyze DNA yield. An in-house method was used to determine mtDNA CN. Comparison of mtDNA CN from nDNA derived calculations and measured mtDNA CN highlighted vast differences. The results emphasize the need to perform qPCR to assess mtDNA CN before NGS analyses of aged bones’ mitogenomes rather than estimating mtDNA CN from nDNA yield to ensure the quality and reliability of the results of NGS analysis.

**Supplementary Information:**

The online version contains supplementary material available at 10.1007/s00414-023-03074-2.

## Introduction

In both routine practice and research in forensic genetics, samples such as hair shafts without roots or skeletonized human remains are frequently encountered that are deemed difficult to analyze because of the poorly preserved state they are found in. This is reflected in nucleic acid forensic analyses, such as gold standard STR typing, for which nDNA from such samples may be lacking in quantity and quality, resulting in the use of alternatives to at least partially find the desired answers [1–4]. The most advantageous of these—despite lacking discriminatory power—is mtDNA [5]. The mitogenome has distinct characteristics such as a round, enclosed shape, making it more resilient to breakdown [6]. The specific inheritance pattern of mtDNA—a mother passes on the mitogenome to all her children, both male and female—makes it possible to examine and follow maternal lineages and populations [7–9]. A unique characteristic of mtDNA is its rather diverse and strongly tissue-dependent copy number (CN) per cell as opposed to DNA, which is always present in two copies per cell [10–14]. Cases in point include crime and missing person identification investigations [15], ancient DNA studies [16], historical case studies [9, 17], and ancestry studies [5].

When badly preserved DNA samples are analyzed, and sequencing of mtDNA must be performed, a correct assessment of the mtDNA copy number is needed, especially when the next-generation sequencing (NGS) approach is used. For NGS, a precise input of nucleic acids is recommended or even required for ruling out factors such as chimeric read and nuclear mitochondrial segments of nDNA called NUMTs, which could impact results and their interpretation, especially when input DNA is too high [18, 19]. According to Parson (W. Parson, personal correspondence, MtDNA analysis, and interpretation using EMPOP, ISFG pre-congress workshop, Prague, September 9th, 2019), 1000 to 5000 mitogenome copies are optimal for the best performance of NGS analysis. For quantifying mtDNA, there are no commercially available kits; this problem was addressed by researchers developing various in-house qPCR mtDNA kits [20–23]. It is possible to follow different published multiplex approaches when preparing an in-house qPCR assay, for example, the one published by Alonso et al. [24]. However, for the widespread use in routine forensic casework, the use of commercial kits is preferred due to the validations required [25–27]. To overcome the problem of the lack of commercial mtDNA qPCR kits, various manuals for using NGS technology for mtDNA sequencing recommend estimation of the input amount of mtDNA based on the nDNA quantity measured. Such an example is the Application Guide for Precision ID mtDNA NGS panels for both the whole genome and control region [28], for which 0.3 ng of DNA is claimed to correspond to 8700 copies of the mitogenome; even so, it is noted that the sample source and degradation may affect this prediction [28]. Because of the tissue specificity of the mitogenome CN, such predictions may be inaccurate to various degrees even if made for a certain type of tissue—the aforementioned poorly preserved bones being one example—which poses a problem for further analyses. However, the recently published Technical Note on the Optimization of Input DNA for Applied Biosystems™ Precision ID mtDNA Panels [29] included commentary advising mtDNA quantification, but no changes have been made in the guidelines for HID Ion Chef™ Instrument automated library preparations.

Generally, the mitogenome CN is tissue-specific [14], and bone tissue requires special attention when determining the correct mtDNA NGS input quantity if one takes into account the findings on the preservation of DNA in old bones. For aged bones, high nDNA degradation has been observed [30, 31], and there is a great variability in the preservation of DNA between different types of bones [31–34] within each bone [35–37] and between individuals [30, 31]. This represents the extraordinary complexity of old bone tissue, which most likely also reflects the differences in mtDNA preservation and its quantity and quality.

This study compares mtDNA CN calculated from nDNA qPCR results ([Auto] fragment (ng/μl) quantity measured with the PowerQuant™ kit (Promega) [38]) based on an NGS panel application guide recommendations [28] and measured mtDNA CN ([113 bp] fragment (copies/μl) measured with an in-house method based on Alonso et al. [24]) to ascertain a possible prediction ability of mtDNA CN from nDNA, using skeletonized human remains excavated from two separate Slovenian Second World War mass graves as a DNA source.

The Slovenian Medical Ethics Committee approved this study, approval number 0120-22/2017/3.

## Materials and methods

### Sample selection

Samples from two mass gravesites located near one another and dating back to the Second World War were selected because of their challenging yet relevant characteristics in forensic genetic examinations (skeletonized human remains, long postmortem interval, and DNA degradation). The samples were the diaphyses of femurs, cut below the greater trochanter, from 75 skeletons. Both gravesites are located in Slovenia, and their characteristics have been described in previously published studies [30, 37].

### Preventing contamination

The utensils were cleaned with 6% sodium hypochlorite, followed by a bidistilled water rinse, 80% ethanol wash, and sterilization. UV irradiation of utensils, plastic consumables, and reagents was the final step in preventing contamination. Single-use nitrile gloves, gowns, and caps were used by personnel to avoid contamination with contemporary DNA. Extraction-negative controls and negative template controls were used to track any possible contamination events.

### Bone sample preparation and automated DNA extraction

A room designed specifically for treatment of skeletonized human remains was used to prepare the samples; contaminants that were possibly present on the surface were removed with the outermost layer of bone in a fume cupboard (Iskra Pio, Šentjernej, Slovenia) by using a rotary tool (Schick, Schemmerhofen, Germany) with detachable burrs and diamond discs. A 5% Alconox detergent (Sigma-Aldrich, St. Louis, MO, USA) wash followed, proceeded by washing with bidistilled water (Millipore, Darmstadt, Germany) and 80% ethanol (Fisher Scientific, Loughborough, UK). The samples were then dried overnight and broken into smaller pieces that were then pulverized; to prevent DNA damage caused by overheating, the metal jars used for grinding were cooled with liquid nitrogen just before grinding the samples into powder with a Bead Beater MillMix 20 homogenizer (Tehtnica, Domel, Železniki, Slovenia). The oscillation frequency was set to 30 Hz and the time of grinding to 1 min. Ten milliliters of ethylenediaminetetraacetic acid (EDTA; Promega, Madison, WI, USA) was added to 0.5 g of fine bone powder to demineralize it through overnight incubation at 37 °C. A previously published research [39] describes this optimized process in extensive detail. The Biorobot EZ1 (Qiagen, Hilden, Germany) and the EZ1 DNA Investigator Kit (Qiagen) were used with the following settings as suggested by the manufacturer: trace protocol and 50 μl of DNA in a Tris EDTA buffer elution.

### DNA quantification

An average of replications was used as a result for each sample, and requantification was performed in samples where more than 2-fold difference between the replicates was observed.

### DNA yield

To analyze DNA, the PowerQuant System (Promega) was used. The kit measures the amount of nDNA [Auto] target, the amount of Y-chromosome DNA, and a longer [Deg] target to assess potential degradation (the ratio between the [Auto]/[Deg] targets) and the presence of PCR inhibitors. The two multi-copy targets for assessing degradation differ in length: the shorter 84 bp autosomal [Auto] target and the longer 294 bp degradation [Deg] target. The presence of PCR inhibitors is determined with internal positive control (IPC), which co-amplifies with the sample. The [Auto]/[Deg] ratio threshold was set to 2, and the IPC shift threshold was set to 0.30, as per the manufacturer’s guidelines. The QuantStudio™ 5 Real-Time PCR System (TFS) and Design and Analysis Software v1.5.2 (TFS) were used. Serial dilutions (0.0032 ng/μl, 0.08 ng/μl, 2 ng/μl, and 50 ng/μl) of PowerQuant® Male gDNA (Promega) with PowerQuant® Dilution Buffer (Promega) were used for the standard curve, as per manufacturer’s guidelines. The PowerQuant Analysis Tool (Promega), a Microsoft Excel macro file provided by the manufacturer, was used to view the results, calculate the results averages, and calculate the Auto/Deg ratio. Quantifications were performed in duplicate for all samples, and a negative template control was included in each run. The quantity of DNA was determined from the shorter [Auto] target, and the [Deg] target was used only for calculating the degradation of DNA [38].

### mtDNA copy number

A modified in-house qPCR protocol was structured in line with the method published by Alonso et al. (2004), in which a 620 bp fragment (isolated from a human donor DNA) covering the HV1 region of mitogenome was used as a standard, and a single-copy target (113 bp) was used to determine the mitogenome copy number [24]. A spectrophotometric method using Synergy H4 (BioTek) was used to quantify the mitogenome. The copy number was then calculated based on the molecular weight of the fragment. After concentration normalization, serial dilutions (6,000,000 copies/μl, 600,000 copies/μl, 60,000 copies/μl, 6000 copies/μl, 600 copies/μl, and 60 copies/μl) were used to create a standard curve. The primers and fluorescent-labeled TaqMan MGB probes were custom-ordered (Applied Biosystems, Renfrewshire, UK). The QuantStudio™ 5 Real-Time PCR System (TFS) and Design and Analysis Software v1.5.2 (TFS) were used for quantification. TaqMan™ Universal Master Mix II with Uracil-N-glycosylase (TFS) was used instead of 1X TaqMan Universal PCR Master Mix (AB) because the latter has been discontinued. Duplicate quantifications were made for all samples, and a negative template control was included in each run.

Additionally, the copy number of mitogenome was calculated as per manufacturer’s guidelines, where it is stated that 0.3 ng of nDNA corresponds to 8700 copies of mitogenome (or 0.1 ng of nDNA is 2900 copies of mitogenome) [28]. Furthermore, a ratio between measured and calculated copy number of mtDNA has been determined.

### Statistical evaluation

IBM SPSS Statistics for Mac, Version 23.0 (IBM Corp., Armonk, NY 2015), and Microsoft Excel for Mac, Version 16.67 (Microsoft Corporation 2022), were used to carry out descriptive statistics.

## Results

**Fig. 1:**
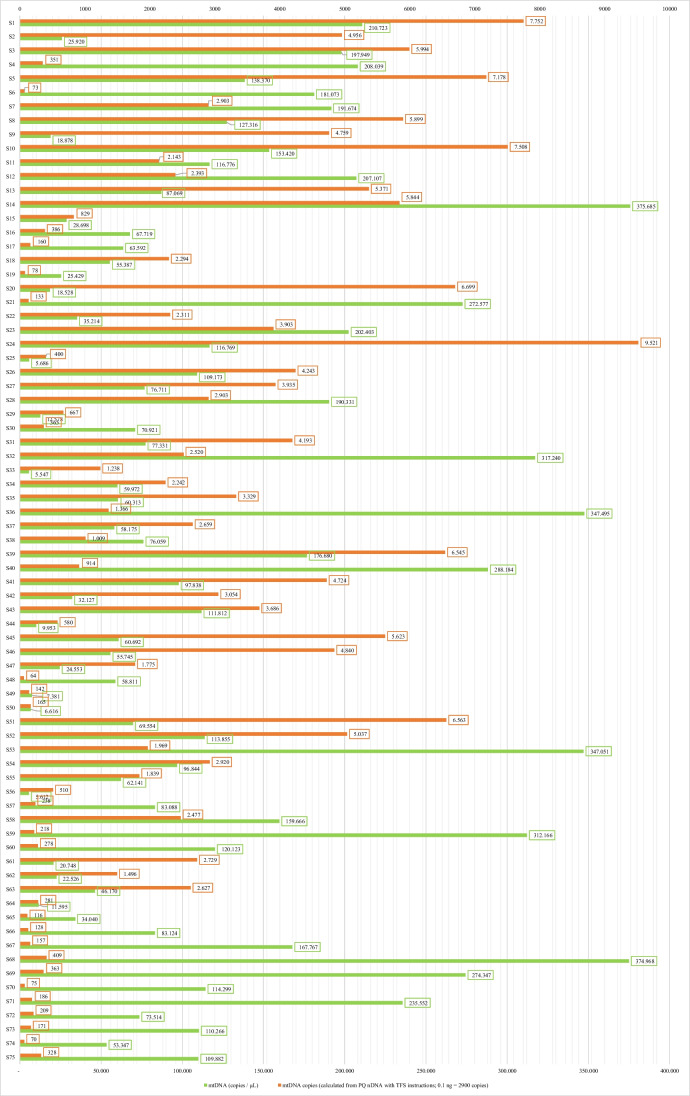
Comparison of qPCR results of mtDNA [113 bp] in copies/μl and calculated mtDNA copies/μl based on nDNA [Auto] target (84 bp) (PowerQuant System, Promega) and manufacturer's instructions [28]

The complete results for 75 femurs excavated from the Second World War mass graves for the nDNA PowerQuant Kit (Promega) qPCR analyses, mtDNA in-house qPCR analyses, and calculations are shown in Supplementary Material 1 (SM1—Table 1, 2, and 3) and in Fig. 1.

### qPCR and calculations

The quantity of nDNA [Auto] fragment expressed in ng/μl ranged between 0.0022 ng/μl and 0.3283 ng/μl, and it averaged 0.0846 ng/μl. IPC shifts were between −3 (S59) and 0.76 (S65) (see SM1, Table 1).

The measured CN of the mtDNA 113 bp fragment (copies/μl) ranged from 5547 to 375,685 copies/μl and averaged 115,019.79 copies/μl (see SM1, Table 1).

The calculated mtDNA CN ranged from 64 to 9521, and the ratio between the measured and calculated CN ranged from 3 to 2498 (see SM1, Table 2).

## Discussion

Our results derived from 75 femurs from two Second World War mass graves showed that there is a vast difference when calculating mtDNA copy number from nDNA quantity (using the short multi-copy target PowerQuant System (Promega), and as recommended by TFS [28]) and mtDNA quantity measured with qPCR (using short in-house single-copy target from quantification assay published by Alonso et al. [24]). When quantifying nDNA with the PowerQuant System (Promega) and mtDNA with Alonso’s in-house assay, shorter targets are used to determine nDNA and mtDNA yields [24, 38], and therefore, these targets (84 bp for nDNA and 113 bp for mtDNA) were used for calculations and comparisons. The length of the shorter mtDNA target also coincides with the length of the targets in NGS applications; for example, 153 bp is the average amplicon size for the Precision ID mtDNA Control Region Panel, as stated in the Precision ID mtDNA Panels with the HID Ion S5™ / HID Ion GeneStudio™ S5 System Application Guide [28], indicating that, for estimating the appropriate input of mtDNA for NGS analysis, information on the quantity of the shorter 113 bp mtDNA fragment is important.

Even if the same type of skeletal element was used, and the same intra-bone part was sampled, the wide interval between minimum and maximum in both targets for measuring the nDNA yield as well as the mtDNA copy number shows large variability in DNA content in samples even though the remains had the same postmortem interval (~75 years) and had been exposed to similar environmental conditions (both mass graves were located in karst caves), indicating the high complexity of aged bone tissue in the preservation of DNA. Namely, numerous studies have shown that there are differences in nDNA yields between different types of skeletal elements [33, 40, 41] and even between different parts of the same skeletal element [35–37]. Furthermore, in all samples measured, mtDNA CN was 3 to 2498-fold larger than from nDNA qPCR results calculated mtDNA CN, which indicates that an underestimation would have been made if mtDNA CN was calculated from nDNA qPCR results instead of being measured using qPCR mtDNA target. This can be attributed to the better resilience [6] and much larger CN of mitogenome in comparison to nDNA [10–14]. Our results conclude that nDNA is much more degraded compared to mtDNA, which certifies that mtDNA has a better preservation ability in aged skeletal remains. Calculating mtDNA CN from nDNA qPCR results as per recommendations [28] therefore portrays much lower mtDNA CN than it would if mtDNA CN is measured with qPCR using mtDNA target. Moreover, variability in DNA content between the samples can also be attributed to inter-individual differences, as seen in our study of Second World War femur diaphyses and other studies performed on aged bones [30, 31].

Successful identification of skeletonized remains often relies upon DNA analyses, frequently focusing on the mid-diaphysis of weight-bearing long bones. In a study that explored intra-bone DNA variability using bovine and porcine femora, along with calcanei and tali, DNA from fresh and short-term environmentally exposed bone was extracted utilizing demineralization and standard lysis buffer protocols. DNA quantity and quality were measured. Overall, femoral epiphyses, metaphyses, and tarsals had more nuclear and mitochondrial DNA than did the femoral diaphyses. DNA loss was much more rapid in buried bones than in surface-exposed bones, while DNA quality differed based on environment but not bone region/element. However, the relation between nDNA yield and mtDNA CN was not explored, bones were of animal origin and either fresh or only short-term exposed to the environment (buried) [42]. To gain insight into specific contexts such as mtDNA copy number in decades-long environmentally exposed human bones, additional analyses must be performed. Because of the high complexity of DNA preservation in old skeletal remains, we recommend performing the mtDNA qPCR analyses before mtDNA NGS, and not assessing it from nDNA qPCR results, to avoid issues that could result from incorrect mtDNA input in high-priced NGS analyses. Low-cost qPCR tests are undemanding and—when working with challenging samples—a necessity.

## Conclusion

According to the comparison between calculated mtDNA CN from nDNA quantity (PowerQuant [Auto] target) and qPCR measured mtDNA CN [113 bp target], there would have been an underestimation at assessing mtDNA CN in the 75 Second World War femurs studied. Because of the great complexity of aged bones, there is a need for mtDNA qPCR quantification for NGS analyses to correctly meet the input requirements of such analyses and to generate accurate results. It must be stressed that the association between nDNA and mtDNA examined in this study is only applicable in this way when comparing the results of the specific kit/method combination: a multi-copy target PowerQuant System (Promega) for nDNA and an in-house single-copy target qPCR mtDNA quantification method based on Alonso et al. [24] using short targets of each assay. Comparisons and relations between any other combinations of kits used for determining the nDNA quantity and mtDNA CN must be tested separately.

### Supplementary information

ESM 1 (XLSX 23 kb)

## Data Availability

The authors declare that all the data are available.
